# Psychological problems among elementary and high school educators in Canada: association with sick days in the prior school year

**DOI:** 10.3389/fpsyg.2024.1442871

**Published:** 2024-10-07

**Authors:** Belinda Agyapong, Yifeng Wei, Raquel da Luz Dias, Ade Orimalade, Pamela Brett-MacLean, Vincent Israel Opoku Agyapong

**Affiliations:** ^1^Department of Psychiatry, Faculty of Medicine and Dentistry, University of Alberta, Edmonton, AB, Canada; ^2^Department of Psychiatry, Faculty of Medicine, Dalhousie University, Halifax, NS, Canada

**Keywords:** burnout, educators, generalized anxiety disorder, moderate depressive disorder, resilience, sick days, stress, teachers

## Abstract

**Background:**

Increased sick leave among educators can detrimentally impact students’ productivity, and academic achievement. It remains unknown whether the number of sick days taken by educators in the preceding school year correlates with the prevalence or severity of psychological problems among educators in the subsequent school year.

**Objective:**

This study aimed to examine the number of self-reported sick days taken by educators in three Canadian provinces during the 2021/2022 academic year and its association with measures of stress, burnout, low resilience, depression, and anxiety during the 2022/2023 school year.

**Methods:**

Data was collected from educators in three Canadian provinces, Alberta, Nova Scotia, and Newfoundland and Labrador, from September 1, 2022, to August 30, 2023. The Maslach Burnout Inventory-Educator Survey (MBI-ES), the Brief Resilience Scale (BRS), and the Perceived Stress Scale were used to assess burnout, resilience, and stress, respectively. Likely Generalized Anxiety Disorder (GAD) and likely Major Depressive Disorder (MDD) were assessed using the Generalized Anxiety Disorder-7 and Patient Health Questionnaire-9 scales, respectively. Statistical analysis was conducted using SPSS version 28.

**Results:**

A total of 763 subscribers completed all the demographic, professional questions, and clinical scales, giving a response rate of 39.91%. Of these, there were 94 (12.3%) males and 669 (87.7%) females. Educators who reported taking 11 or more sick days in the previous academic year were at least three times more likely to exhibit high stress, emotional exhaustion, likely GAD, low resilience, and likely MDD than educators with no sick days during the preceding year. Similarly, educators with 11 or more sick days had significantly higher mean scores on the GAD-7 scale, the PHQ-9 scale, the PSS-10, the MBI Emotional Exhaustion subscale, and the MBI Depersonalization subscale than those with zero sick days.

**Conclusion:**

This study demonstrates a significant association between sick days and the prevalence and severity of high stress, low resilience, burnout, anxiety, and depression among educators. Short-term sick leave can escalate into long-term absences without adequate support for teachers. Governments and policymakers in the education sector must foster a supportive environment that enables teachers to thrive and effectively perform their professional role without taking prolonged sick days, which can undermine student learning and achievement.

## 1 Introduction

While teaching is inherently rewarding, it has unique challenges that can contribute to notable psychological health concerns. The responsibilities of educators are far-reaching, encompassing multifaceted roles that often extend beyond academics (Cox, 2020). For example, as teachers perform their role, they engage with colleagues, parents, and caregivers of students, as well as students, both within and outside the classroom. Yet, despite their pivotal role in shaping future generations, teachers are often under-compensated compared to professionals in other fields, with significant disparities in salary distribution across different countries (Mizala and Ñopo, 2016). With a modest starting salary of $55,910, Canadian teachers are unsung heroes shaping the minds and character of the nation’s future generations (Statistics Canada, 2023). These comparatively low wages impact teachers’ financial stability, job satisfaction, and morale. In 2021/2022, Canada’s average gross clinical payment per physician was $357,000, although most physicians are fee-for-service (independent contractors) (Canadian Institute for Health Information, 2022). With increasing work demands (such as larger class sizes, administrative responsibilities, expectations for standardized testing, etc.), this disparity between their dedicated efforts and rewards can exacerbate stress and burnout among teachers (Maslach and Leiter, 2016).

Research indicates that teachers may experience higher rates of psychological disorders compared to other professions and are at higher risk of workplace stress, leading to burnout, depression, and other psychosomatic symptoms (Schonfeld et al., 2017). The stress experienced by teachers can be linked to three major overlapping problems: burnout, anxiety, and depression, all of which profoundly impact their health, well-being, effectiveness, or productivity (Agyapong et al., 2022). The COVID pandemic exacerbated these challenges as educators transitioned to online instruction. Lack of adequate administrative support and resulting anxiety were significant predictors of teacher burnout during the COVID-19 pandemic (Pressley, 2021). A survey of mental health professionals reported that the majority characterized burnout as a state of exhaustion, which was viewed as a risk factor for developing a mental disorder (Hamann et al., 2013). Similarly, a study conducted during the second wave of the pandemic found high rates of depression, anxiety, and stress among teachers (67, 73, and 86%, respectively) (Lizana and Lera, 2022).

The demanding nature of teaching often takes a toll on educators’ mental health, resulting in the need for sick leave to recuperate, which affects not only teachers but also carries significant social and economic costs for students, educational organizations, and society as a whole (Carlsen, 2012). In the U.K., a survey by the Department of Education reported that 40,000 pre-retirement teachers resigned from state schools during the 2021/2022 academic year (Adams, 2023). This survey also documented a notable increase in teacher absenteeism due to illness, with teachers accumulating over 3 million sick days during the 2021/2022 school year, marking a surge of more than 50% compared to pre-pandemic levels in 2018/2019 academic year (Adams, 2023). Data from general practitioners also reported that 81% of mental health issues among the general workforce were associated with sickness absence (Hussey et al., 2012). Stress at work has also been linked to poor well-being and increased sickness leave due to depressive symptoms (Kidger et al., 2016).

Similarly, the increasing severity of depression has been associated with increased sick absences. The outcome of a study among workers in the U.S. reported that all levels of depression were linked to decreased work productivity, and absenteeism was significantly positively associated with the severity of depression (Jain et al., 2013). While there was an uptick in sick days taken by civil servants in 2022 due to stress and other mental health issues, this increase was comparatively lower than that of teachers (Campbell, 2024).

Teachers reported an absence rate of 19%, with less experienced teachers exhibiting higher rates of absence (Usman et al., 2007). Similarly, secondary school teachers have identified burnout (specifically emotional exhaustion) and job dissatisfaction as significant factors associated with psychiatric sick leave (Antonio Moriana and Herruzo, 2006). Further, elevated levels of emotional exhaustion were associated with long-term sickness absence, while low levels of professional efficacy (P.E.) did not show such a correlation (Salvagioni et al., 2022). In Canada, a survey conducted among members of the British Columbia Teachers’ Federation in 2023 revealed heightened stress and workload among nearly two-thirds of the respondents, with approximately 40% reporting a decline in their physical or mental health compared to the previous year (British and Columbia’s Teachers' Federation, 2023). Another Canadian survey found that more than 25% of teacher respondents had taken a leave of absence, with stress being the primary reason cited (Ferguson et al., 2022). This study also found that the stress teachers experience at school is frequently due to factors like increased workload and lack of support from the administration, which invariably adversely impact educators’ home lives.

Teachers exhibited a 6% higher rate of sick leave compared to other professionals, such as physiotherapists and occupational therapists, resulting in a loss of 3.3% of available teacher time due to sickness absence (Bowers and McIver, 2000). While sickness absenteeism is commonly associated with physical illness (Jansson and Alexanderson, 2013; Winkler, 1980), its profound connection to mental well-being among teachers may often be overlooked. Again, limited Canadian research focuses on the association between educators’ sick days and their psychological well-being. Further, what is driving the rise in sickness absences among teachers needs further exploration. Thus, the current study aims to evaluate the self-reported number of sick days taken by teachers during a school year and their association with the prevalence and severity of high stress, burnout, low resilience, anxiety, and depression during the subsequent school year.

This study aimed to achieve the following objectives:

- Assess whether the number of sick days teachers take in Alberta, Nova Scotia, Newfoundland, and Labrador during the 2021/2022 school year predicts high stress levels among educators.
- Examine whether the number of sick days taken by teachers in these provinces during the same period predicts the occurrence of burnout among educators.
- Investigate whether the number of sick days teachers take in the three provinces during the same period predicts low resilience among educators.
- Determine if the number of sick days teachers take in the three provinces during the same period predicts the likelihood of Generalized Anxiety Disorder (GAD) among educators.
- Explore whether the number of sick days taken by teachers in the three provinces during the same period predicts the likelihood of Major Depressive Disorder (MDD) among educators.

### 1.1 Hypothesis

We hypothesize that the number of sick days taken by educators during the 2021/2022 academic year will be positively associated with the prevalence and severity of high stress, burnout, low resilience, and the likelihood of GAD and MDD in the subsequent school year.

## 2 Methodology

### 2.1 Study design

This is an exploratory study that utilized a cross-sectional survey design. Secondary analysis was performed on baseline survey data from the Wellness4Teachers program. Data were collected using web-based questionnaires distributed via a link to participants’ mobile phones. The questionnaires were administered using the University of Alberta REDCap platform, a secure web application designed to create and manage online surveys (REDCap, 2022).

### 2.2 Study settings

In 2024, Canada’s population reached 40,769,890 individuals (Statistic Canada, 2024). Nova Scotia, Newfoundland and Labrador, and Alberta, where the study was conducted, are three of Canada’s 13 provinces and territories (Government of Canada, 2024). The Canadian Teachers’ Federation is a national alliance of provincial and territorial member organizations representing over 232,000 teachers across the country (Canadian Teachers' Federation, 2017).

Nova Scotia, Newfoundland, and Labrador are both eastern provinces of Canada, with populations estimated at 1,066,416 and 540,418, respectively (Statistics Canada, 2023). The Nova Scotia Teachers Union aims to improve the quality of public education and represents more than 10,000 public school teachers, community college faculty, professional support staff, and educators working in the Atlantic Provinces Special Education Authority (Nova Scotia Teachers Union, 2015). In Newfoundland and Labrador, the English School District is the school board that oversees all English-language primary and secondary education, with approximately 63,000 students across 250 schools and more than 10,000 staff members (Newfoundland and a.L.E.S. District, District Overview, 2022). There are 5,232 full-time equivalent teachers in Newfoundland and Labrador’s 2023/2024 academic year (Government of Newfoundland and Labrador, 2024).

Alberta, located in Western Canada, has an estimated population of 4,756,408 and about 32,523 full and part-time teachers (Statistics Canada, 2023; The Alberta Teachers' Association, 2023). According to 2002/2023 data collected from the Canadian Teachers’ Federation (CTF) survey, 72 percent of the members of teacher organizations in Canada are women, and 28 percent are men (O’Haire, 2024). This shows some degree of gender imbalance in the teaching profession, with 63% of teachers being female in Alberta (Schwab, 2019). 84% of elementary school and kindergarten teachers in Nova Scotia are female (Government of Canada, 2024), and over two-thirds (68.7%) of teachers in 2007/08 were female in Newfoundland and Labrador (Government of Newfoundland and Labrador, 2008).

### 2.3 Data collection

Data collection commenced during the 2022/2023 academic year through the Wellness4Teachers program, which was initiated at the beginning of the academic year (Agyapong et al., 2022). This program provides teachers with one-way (non-interactive) psychological intervention text messages based on cognitive behavioral therapy principles, delivered to their mobile phones upon signing up.

#### 2.3.1 Participants

Teachers in Newfoundland and Labrador, Nova Scotia, and Alberta were invited to self-subscribe to the program (Agyapong et al., 2022), facilitated by collaborative efforts with the Alberta Teachers Association, the Alberta Catholic School Boards, the Nova Scotia Teachers Union, and the Newfoundland and Labrador Teachers Union. Teachers subscribed by texting “TeachWell” to a designated phone number, which automatically registered them to receive supportive text messages (Agyapong et al., 2022). Upon enrollment, participants were invited to complete a web-based survey via a link in the welcome text message. The baseline survey collected demographic information (age, sex, ethnicity, marital and housing status), professional details (the total number of sick days teachers took in the preceding year, years of teaching experience, class size, major role, area of specialization, and primary stressors), and clinical variables; stress, burnout, resilience, anxiety, and depression. Teachers responded to the question: “As far as you can recall, in the 2021/2022 school year, how many sick days did you take off school?” Typically, the survey took 10–15 min to complete. Completion of the baseline survey was entirely voluntary, irrespective of receiving daily supportive text messages.

### 2.4 Outcome measures

The primary outcome measures in this study are the measures of association between the number of sick days taken by the educators and the prevalence of high stress, burnout, low resilience, anxiety, and depression during the subsequent school year. The secondary outcome measures included measures of association between the number of sick days taken by the educators and the severity of high stress, burnout, low resilience, anxiety, and depression during the subsequent school year.

#### 2.4.1 Instruments

The Maslach Burnout Inventory-Educator Survey (MBI-ES) (Maslach et al., 2022; Maslach et al., 1997) was used to collect teacher burnout data. The MBI-ES is a 22-item instrument that measures emotional exhaustion, depersonalization, and personal accomplishment. For each of the three dimensions, emotional exhaustion scores ≥27 were deemed high, 17–26 were deemed normal, and 0–16 were deemed low. Similarly, for depersonalization scores, 13 and over was deemed high, 7–12 was deemed normal, and 0–6 was deemed low. Finally, 0–31 was deemed high for personal accomplishment, 32–38 was deemed normal, and 39 and over was deemed low. An analysis of the internal consistency of the MBI-ES questionnaire yielded a Cronbach’s alpha of 0.785 for the full scale, which indicated scale reliability. For the emotional exhaustion subscale, the alpha coefficient was 0.93, suggesting excellent reliability, while the alpha coefficient for the depersonalization subscale was 0.618, suggesting questionable reliability, and for the personal accomplishment subscale, the alpha coefficient was 0.776, indicating satisfactory reliability (Vukmirovic et al., 2020).

Resilience was measured using the Brief Resilience Scale (BRS; mean scores of 1.00–2.99 suggest low resilience, 3.00–4.30 suggest normal resilience, and 4.31–5.00 suggest high resilience) (Smith et al., 2008). The Perceived Stress Scale 10 (PSS-10; a score of ≥27 indicates likely high stress) was used to measure stress (Cohen et al., 1983). Again, a PSS-10 score of ≤13 indicates low stress, scores of 14 to 26 indicate moderate stress, and scores of ≥27 indicate high stress. The BRS and the PSS-10 have Cronbach’s *α* of 0.78 and 0.82, respectively, suggesting good internal reliability (Soer et al., 2019; Remor, 2006).

Likely Generalized Anxiety Disorder (GAD) among participants was evaluated with the Generalized Anxiety Disorder-7 (GAD-7) scale (Spitzer et al., 2006). This scale consists of seven self-reported items defining the symptoms of GAD. Participants with a GAD-7 score ≥ 10 were considered to have moderate to high anxiety or likely GAD symptoms, and those with GAD-7 scores <10 were considered to have low anxiety (GAD unlikely) (Johnson et al., 2019). Cronbach’s alpha for the GAD-7 scale when used in primary care was 0.92, and the split-half reliability was 0.82, revealing a good level of reliability (Ahmad et al., 2017).

Likely, Major Depressive Disorder (MDD) was measured with the Patient Health Questionnaire-9 (PHQ-9). PHQ-9 score ≥ 10 indicates moderate to severe depression or likely MDD (Kroenke et al., 2001), whereas PHQ-9 score of <10 signifies at most mild depression (MDD unlikely). The Cronbach’s *α* for PHQ-9 reported in one study was 0.74, demonstrating adequate internal consistency (Titov et al., 2011). The MBI-ES, BRS and PSS-10, PHQ-9, and GAD-7 scales were studied as categorical variables for prevalence estimates and as means for severity estimates for the psychological conditions.

### 2.5 Statistical analysis

Data analysis was conducted using SPSS Version 28 (IBM, 2022). The variable “Sick days” was categorized into five groups: “No sick days,” “1–5 sick days,” “6–10 sick days,” “11–15 sick days,” and “16 or more sick days.” Seven separate multivariate binomial logistic regression analyses assessed the independent association between sick days and psychological problem variables (high stress, emotional exhaustion, depersonalization, lack of personal accomplishment, likely MDD, likely GAD, and low resilience).

Each regression model included demographic and work-related variables, including sick days, as predictors. Prior to regression analyses, correlational analysis was performed to eliminate strong intercorrelations (Spearman’s correlation coefficient of 0.7 to 1.0 or – 0.7 to – 1.0) among predictor variables. Odds ratios (OR) with two-tailed significance (*p* ≤ 0.05) and confidence intervals were used to determine the effect of the “Sick days” variable on respondents’ self-reported psychological conditions while controlling for other variables in the model.

To compare the severity of the psychological conditions under study, a one-way analysis of variance (ANOVA) with two-tailed significance (*p* ≤ 0.05) was used to evaluate differences in mean scores across the five categories of the “sick days” variable. Post-hoc analysis was conducted using the HSD test. In cases where homogeneity of variance was violated, Welch’s *F*-test and the Games-Howell *post-hoc* test were planned to be used.

## 3 Results

Table 1 summarizes the demographic, work-related, and professional characteristics of participants based on the number of sick days. Of the 1912 teachers who subscribed to the Wellness4Teachers program, 763 subscribers completed all the demographic, professional questions, and clinical scales, giving a response rate of 39.91%. Of those included in the study, a total of 535 (70.1%) live in Alberta, 127 (16.6%) live in Nova Scotia, and 101 (13.2%) live in Newfoundland and Labrador. Overall, there were 94 (12.3%) males and 669 (87.7%) females, and most were Caucasians (692, 90.7%). A total of 608 (79.7%) of participants taught in public schools and 140 (18.3%) in Catholic schools. Most participants, 290 (38.0%), had 20 years or less but more than 10 years of teaching experience, and 228 (29.9%) had more than 20 years of teaching experience.

**Table 1:** Frequency distribution of demographic, work-related, and professional variables based on participants’ number of sick days absences.

Variables	Zero sick days *N* = 54 n (%)	1–5 sick days *N* = 255 n (%)	6–10 sick days *N* = 235 n (%)	11–15 sick days *N* = 89 n (%)	16 or more sick days *N* = 130 n (%)	Chi^2^/Fisher’s exact^*^ test values	*p*-value	Total *N* = 763 n (%)
**Sociodemographic characteristics**								
Age (years)								
18–25	4 (16.0%)	10 (40.0%)	8 (32.0%)	2 (8.0%)	1 (4.0%)	11.09	0.52	25 (3.3%)
26–40	22 (7.6%)	92 (31.9%)	94 (32.6%)	34 (11.8%)	46 (16.0%)			288 (27.7%)
41–60	27 (6.3%)	144 (33.6%)	125 (29.1%)	52 (12.1%)	81 (18.9%)			429 (56.2%)
61 and above	1 (4.8%)	9 (42.9%)	8 (38.1%)	1 (4.8%)	2 (9.5%)			21 (2.8%)
Sex at birth								
Male	16 (17.0%)	37 (39.4%)	24 (25.5%)	11 (11.7%)	6 (6.4%)	24.22	<0.001	94 (12.3%)
Female	38 (5.7%)	218 (32.6%)	211 (31.5%)	78 (11.7%)	124 (18.5%)			669 (87.7%)
Relationship status								
Single	15 (13.3%)	42 (37.2%)	31 (27.4%)	12 (10.6%)	13 (11.5%)	23.6	0.10	113 (14.8%)
Married	28 (5.7%)	167 (33.9%)	159 (32.3%)	52 (10.6%)	86 (17.5%)			492 (64.5%)
Common- law or partnered	7 (6.9%)	33 (32.4%)	31 (30.4%)	15 (14.7%)	16 (15.7%)			102 (13.4%)
Separated or divorced	3 (6.4%)	12 (25.5%)	13 (27.7%)	7 (14.9%)	12 (25.5%)			47 (6.2%)
Other	1 (11.1%)	1 (11.1%)	1 (11.1%)	3 (33.3%)	3 (33.3%)			9 (1.2%)
Number of children								
No child	23 (9.7%)	85 (35.9%)	74 (31.2%)	25 (10.5%)	30 (12.7%)	12.47	0.71	237 (31.1%)
One child	6 (5.3%)	38 (33.6%)	31 (27.4%)	16 (14.2%)	22 (19.5%)			113 (14.8%)
Two children	16 (5.4%)	92 (31.2%)	92 (31.2%)	36 (12.2%)	59 (20.0%)			295 (38.7%)
Three children	5 (6.0%)	27 (32.5%)	27 (32.5%)	8 (9.6%)	14 (16.9%)			83 (10.9%)
Four or more children	4 (11.4%)	11 (31.4%)	11 (31.4%)	4 (11.4%)	5 (14.3%)			35 (4.6%)
Ethnicity								
Indigenous	1 (5.9%)	8 (47.1%)	2 (11.8%)	3 (17.6%)	3 (17.6%)		0.06	17 (2.2%)
African descendants	1 (16.7%)	2 (33.3%)	2 (33.3%)	1 (16.7%)	0 (0.0%)			6 (0.8%)
East Asian	3 (25%)	4 (33.3%)	2 (16.7%)	0 (0.0%)	3 (25.0%)	40.23		12 (1.6%)
Latino	2 (22.2%)	3 (33.3%)	4 (44.4%)	0 (0.0%)	0 (0.0%)			9 (1.2%)
Middle Eastern	2 (40.0%)	1 (20.0%)	0 (0.0%)	0 (0.0%)	2 (40.0%)			5 (0.7%)
South Asian	0 (0.0%)	6 (75.0%)	1 (12.5%)	0 (0.0%)	1 (12.5%)			8 (1.0%)
Caucasian (European descent)	44 (6.4%)	228 (32.9%)	218 (31.5%)	84 (12.1%)	118 (17.1%)			692 (90.7%)
Other ethnicities	1 (7.1%)	3 (31.4%)	6 (42.9%)	1 (7.1%)	3 (21.4%)			14 (1.8%)
Housing status								
Own home	39 (6.1%)	211 (33.2%)	202 (31.8%)	76 (11.9%)	108 (17.0%)	8.73	0.34	636 (83.4%)
Rented accommodation	12 (11.3%)	35 (33.0%)	29 (27.4%)	10 (9.4%)	20 (18.9%)			106 (13.9%)
Live with family or friends	3 (14.3%)	9 (42.9%)	4 (19.0%)	3 (14.3%)	2 (9.5%)			21 (2.8%)
Province of residence								
Alberta	42 (7.9%)	202 (37.8%)	151 (28.2%)	60 (11.2%)	80 (15.0%)	22.42	0.004	535 (70.1%)
Nova Scotia	5 (3.9%)	32 (25.2%)	49 (38.6%)	14 (11.0%)	27 (21.3%)			127 (16.6%)
Newfoundland and Labrador	7 (6.9%)	21 (20.8%)	35 (34.7%)	15 (14.9%)	23 (22.8%)			101 (13.2%)
**Work-related variables**								
Location of school								
Rural setting	14 (4.7%)	95 (32.2%)	103 (34.9%)	31 (10.5%)	52 (17.6%)	7.2%	0.13	295 (38.7%)
Urban setting	40 (8.5%)	160 (34.2%)	132 (28.2%)	58 (12.4%)	78 (16.7%)			468 (61.3%)
Area of teaching specialization								
English	9 (7.1%)	45 (35.4%)	37 (29.1%)	15 (11.8%)	21 (16.5%)	0.000^*^	0.00^*^	127 (16.6%)
Mathematics	9 (16.7%)	18 (32.7%)	18 (32.7%)	3 (5.5%)	7 (12.7%)			55 (7.2%)
Sciences (physics, chemistry, biology)	8 (15.4%)	21 (40.4%)	9 (17.3%)	4 (7.7%)	10 (19.2%)			52 (6.8%)
Arts (history, geography, social studies, etc.)	5 (7.1%)	31 (44.3%)	18 (25.7%)	10 (14.3%)	6 (8.6%)			70 (9.2%)
Music	0 (0.0%)	5 (21.7%)	9 (39.1%)	3 (13.0%)	6 (26.1%)			23 (3.0%)
Physical education	3 (15.0%)	6 (30.0%)	4 (20.0%)	5 (25.0%)	2 (10.0%)			20 (2.6%)
Other	20 (4.8%)	129 (31.0%)	140 (33.7%)	49 (11.8%)	78 (18.8%)			416 (54.5%)
Teach only in your area of specialization								
No	34 (7.8%)	149 (34.0%)	132 (30.1%)	50 (11.4%)	73 (16.7%)	1.08	0.89	438 (57.4%)
Yes	20 (6.2%)	106 (32.6%)	103 (31.7%)	39 (12.0%)	57 (17.5%)			325 (42.6%)
Number of years teaching								
5 years or less	15 (14.0%)	41 (38.3%)	34 (9.3%)	10 (9.3%)	7 (6.5%)	30.54	0.002	107 (14.0%)
10 years or less but more than 5 years	10 (7.2%)	38 (27.5%)	48 (34.8%)	23 (16.7%)	19 (13.8%)			138 (18.1%)
20 years or less but more than 10 years	11 (3.8%)	103 (35.5%)	89 (30.7%)	31 (10.7%)	56 (19.3%)			290 (38.0%)
More than 20 years.	18 (7.9%)	73 (32.0%)	64 (28.1%)	25 (11.0%)	48 (21.1%)			228 (29.9%)
Class size taught								
20 or less	11 (7.0%)	52 (32.9)	53 (33.5%)	16 (10.1%)	26 (16.5%)	2.66	0.95	158 (20.7%)
21–27	28 (7.0%)	129 (32.3%)	124 (31.1%)	46 (11.5%)	72 (18.0%)			399 (52.3%)
28 or more	15 (7.3%)	74 (35.9%)	58 (28.2%)	27 (13.1%)	32 (15.5%)			206 (27.0%)
School institution (type)								
Catholic school	16 (11.4%)	51 (36.4%)	37 (26.4%)	16 (11.4%)	20 (14.3%)	14.32	0.07	140 (18.3%)
Public school	35 (5.8%)	199 (32.7%)	192 (31.6%)	73 (12.0%)	109 (17.9%)			608 (79.7%)
Other	3 (20.0%)	5 (33.3%)	6 (40.0%)	0 (0.0%)	1 (6.7%)			15 (2.0%)
Major role								
Elementary school teacher	14 (4.1%)	102 (30.2%)	120 (35.5%)	34 (10.1%)	68 (20.1%)	34.79	0.00	338 (44.3%)
Junior high school teacher	13 (9.6%)	42 (30.9%)	36 (26.5%)	18 (13.2%)	27 (19.9%)			136 (17.8%)
Senior high school teacher	10 (8.8%)	43 (38.1%)	31 (27.4%)	15 (13.3%)	14 (12.4%)			113 (14.8%)
Support staff	1 (3.1%)	11 (34.4%)	8 (25.0%)	7 (21.9%)	5 (15.6%)			32 (4.2%)
Administrator	8 (11.9%)	32 (47.8%)	18 (26.9%)	5 (7.5%)	4 (6.0%)			67 (8.8%)
Other	8 (10.4%)	25 (32.5%)	22 (28.6%)	10 (13.0%)	12 (15.6%)			77 (10.1%)
Source of stress								
Workload	28 (6.6%)	141 (33.4%)	130 (30.8%)	57 (13.5%)	66 (15.6%)	13.74	0.62	422 (55.3%)
Student behavior	13 (8.1%)	53 (33.1%)	47 (29.4%)	18 (11.3%)	29 (18.1%)			160 (21.0%)
Class size	4 (9.5%)	10 (23.8%)	16 (38.1%)	4 (9.5%)	8 (19.0%)			42 (5.5%)
Lack of support from the school administration	4 (6.8%)	17 (28.8%)	18 (30.5%)	8 (13.6%)	12 (20.3%)			59 (7.7%)
Other	5 (6.3%)	34 (42.5%)	24 (30.0%)	2 (2.5%)	15 (18.8%)			80 (10.5%)
**Psychological variables**								
High stress								
No	46 (8.2%)	200 (35.7%)	174 (31.1%)	56 (10.0%)	80 (15.0%)	17.34	<0.001	560 (73.4%)
Yes	200 (35.7%)	55 (27.1%)	61 (30.0%)	33 (16.3%)	46 (22.7%)			203 (26.6%)
Low resilience								
No	33 (7.3%)	172 (38.2%)	137 (30.4%)	53 (11.8%)	5 (12.2%)	24.17	<0.001	450 (59.9%)
Yes	19 (6.3%)	79 (26.2%)	95 (31.6%)	34 (11.3%)	74 (24.6%)			301 (40.1%)
Emotional exhaustion								
No	20 (11.4%)	74 (42.0%)	45 (25.6%)	14 (8.0%)	23 (13.1%)	17.88	0.001	176 (23.1%)
Yes	34 (5.8%)	181 (30.8%)	190 (32.4%)	75 (12.8%)	107 (18.2%)			587 (76.9%)
Depersonalization								
No	43 (7.4%)	206 (35.2%)	181 (30.9%)	61 (10.4%)	94 (16.1%)	7.37	0.12	585 (76.7%)
Yes	11 (6.2%)	47 (29.5%)	54 (30.3%)	28 (15.7%)	36 (20.2%)			178 (23.3%)
Lack of professional accomplishment								
No	40 (7.6%)	182 (34.5%)	155 (29.4%)	60 (11.4%)	90 (17.1%)	2.45	0.65	527 (69.1%)
Yes	14 (5.9%)	73 (30.9%)	80 (33.9%)	29 (12.3%)	40 (16.9%)			236 (30.9%)
Likely GAD								
No	31 (7.6%)	158 (38.5%)	124 (30.2%)	37 (9.0%)	60 (14.6%)	15.5	0.004	410 (54.0%)
Yes	23 (6.6%)	95 (27.2%)	111 (31.8%)	50 (14.3%)	70 (20.1%)			349 (46.0%)
Likely MDD								
No	28 (8.3%)	141 (41.7%)	98 (29.0%)	31 (9.2%)	40 (11.8%)	27.26	<0.001	338 (44.3%)
Yes	26 (6.1%)	114 (26.8%)	137 (32.2%)	58 (13.6%)	90 (21.2%)			425 (55.7%)

### 3.1 Multivariate binary logistic regression analyses

Since there were no strong correlations between the variables under study, all 15 sociodemographic and work-related variables in Table 1 and the “Sick days” variable were included in each of the seven multivariate binary regression models predicting the psychological conditions. Results are outlined in Table 2.

- The model predicting the High Stress was statistically significant; *Χ*^2^(df = 51; *n* = 763) = 96.87, *p* < 0.00, explained between 11.9% (Cox and Snell *R*^2^) and 17.4% (Nagelkerke *R*^2^) of the variance; and correctly classified 74.7% of the cases.
- The model predicting Emotional Exhaustion was statistically significant; *Χ*^2^(df = 51; *n* = 763) = 102.42, *p* < 0.00, explained between 12.6% (Cox and Snell *R*^2^) and 19.0% (Nagelkerke *R*^2^) of the variance; and correctly classified 79.0% of the cases.
- The model predicting depersonalization was statistically significant; *Χ*^2^(df = 51; *n* = 763) = 129.53, *p* < 0.00, explained between 15.6% (Cox and Snell *R*^2^) and 23.6% (Nagelkerke *R*^2^) of the variance; and correctly classified 77.7% of the cases.
- The model predicting lack of personal accomplishment was statistically significant; *Χ*^2^(df = 51; *n* = 763) = 60.35, *p* < 0.00, explained between 7.6% (Cox and Snell *R*^2^) and 10.7% (Nagelkerke *R*^2^) of the variance; and correctly classified 71.7% of the cases.
- The model predicting low resilience was statistically significant; *Χ*^2^(df = 51; *n* = 751) = 119.03, *p* < 0.00, explained between 14.7% (Cox and Snell *R*^2^) and 19.8% (Nagelkerke *R*^2^) of the variance; and correctly classified 67.6% of the cases.
- The model predicting the likely GAD was statistically significant; *Χ*^2^(df = 51; *n* = 759) = 117.00, *p* < 0.00, explained between 14.3% (Cox and Snell *R*^2^) and 19.1% (Nagelkerke *R*^2^) of the variance; and correctly classified 67.1% of the cases.
- The model predicting the likely MDD was statistically significant; *Χ*^2^(df = 51; *n* = 763) = 97.40, *p* < 0.00, explained between 12.0% (Cox and Snell *R*^2^) and 16.0% (Nagelkerke *R*^2^) of the variance, and correctly classified 64.9% of the cases.

**Table 2:** Summary from four multivariate regression models outputs showing the “sick days” variable as a predictor of high stress, emotional exhaustion, depersonalization, and lack of personal accomplishment.

Characteristics	High stress			Emotional exhaustion			Depersonalization			Lack of personal accomplishment		
	OR	95% CI	*P*-value	OR	95% CI	*P*-value	OR	95% CI	*P*-value	OR	95% CI	*P*-value
Sick days variable												
Zero sick days			0.005			0.001			0.01			0.408
1–5 sick days	1.54	0.64–3.68	0.333	1.65	0.80–3.37	0.173	1.25	0.54–2.90	0.61	1.34	0.64–2.82	0.438
6–10 sick days	1.88	0.78–4.53	0.162	3.16^*^	1.48–6.75	0.003	2.18^*^	0.92–5.16	0.08	1.85	0.87–3.93	0.110
11–15 sick days	3.47^*^	1.34–8.96	0.010	3.96^*^	1.60–9.84	0.003	3.00^*^	1.17–7.69	0.02	1.73	0.74–4.03	0.204
16 or more sick days	3.22^*^	1.29–8.03	0.012	3.44^*^	1.47–8.05	0.004	2.69*	1.08–6.69	0.03	1.63	0.73–3.66	0.237

OR, Odds ratio; C.I., confidence interval. *Significance at *p* ≤ 0.05.

Tables 2, 3 show the results of the regression analyses, highlighting the variable of interest, “Sick days,” across the seven regression models predicting high stress, emotional exhaustion, depersonalization, and lack of personal accomplishment, as well as likely MDD, likely GAD, and low resilience.

**Table 3:** Summary from three multivariate regression model outputs showing the “sick days” variable as a predictor of low resilience, likely GAD, and likely MDD.

Characteristics	Low resilience			Likely GAD			Likely MDD		
	OR	95% CI	*P*-value	OR	95% CI	*P*-value	OR	95% CI	*P*-value
Sick days as a variable									
Zero sick days			<0.001			0.001			<0.001
1–5 sick days	0.89	0.434–1.81	0.737	0.891	0.45–1.76	0.74	0.96	0.500–1.86	0.912
6–10 sick days	1.34	0.647–2.76	0.434	1.39	0.70–2.79	0.35	1.79	0.913–3.50	0.090
11–15 sick days	1.31	0.579–2.95	0.519	2.36^*^	1.08–5.16	0.03	2.76^*^	1.276–5.98	0.010
16 or more sick days	2.88^*^	1.319–6.27	0.008	2.01	0.95–4.25	0.07	3.35^*^	1.591–7.05	0.001

OR, Odds ratio; C.I, confidence interval; *Significance at *p* ≤ 0.05.

Table 2 indicates that, after controlling for all demographic and work-related variables, educators who experienced 11–15 sick days and those with 16 or more sick days were approximately three and a half times and three times more likely to present with high stress, respectively, compared to those with no sick days in the preceding academic year. Similarly, educators who had 11–15 sick days and those with 16 or more sick days were four times and three and half times more likely to display emotional exhaustion, respectively, compared to those with no sick days in the preceding academic year. Also, educators with 6–10 sick days, 11–15 sick days, and 16 or more sick days were about two times, three times, and two and a half times more likely to present with depersonalization, respectively, compared with educators who had no sick days in the preceding year. No significant differences were observed between educators with no sick days and those who had sick days in the previous year regarding lack of personal accomplishment when controlling for all sociodemographic and work-related variables.

The results in Table 3 suggest that, even after controlling for all demographic and work-related variables, teachers with 16 or more sick days in the preceding academic year were roughly three times more likely to present with low resilience compared with teachers who had no sick days during the same period. Similarly, teachers who reported 11–15 sick days in the preceding academic year were three times more likely to display symptoms indicative of likely GAD than those with no sick days. Additionally, teachers with 11–15 sick days and those with 16 or more sick days in the previous academic year were about three times more likely to present with symptoms suggestive of likely MDD than teachers with no sick days during the same period.

### 3.2 Analysis of variance

As statistical analysis revealed, there was no violation of the homogeneity of variance for the mean scores across all the psychological conditions under study, so the F test and HSD test for *post hoc* comparisons were used in the ANOVA. The results of both the ANOVA and *post hoc* tests, which compare the mean differences in scores across the various scales for educators who had zero sick days and those with varying sick days, are summarized in Tables 4, 5, respectively.

**Table 4:** ANOVA output comparing the mean scores on standardized rating scales for psychological problems among educators against the number of sick days taken in the preceding school year.

Dependent variable		N	Mean	SD	*F*	*p*-value
GAD-7 score	Zero sick days	54	8.76	5.66	6.825	<0.001
	1–5 days	253	8.53	5.51		
	6–10 days	235	9.37	5.35		
	11–15 days	87	11.17	6.00		
	16 or more days	130	11.08	5.51		
	Total	759	9.54	5.61		
PHQ − 9 score	Zero sick days	54	10.28	6.07	7.977	<0.001
	1–5 days	255	9.71	5.64		
	6–10 days	235	11.21	5.73		
	11–15 days	89	12.52	6.12		
	16 or more days	130	12.80	6.00		
	Total	763	11.07	5.93		
BRS score	Zero sick days	52	3.16	0.83	9.295	<0.001
	1–5 days	251	3.32	0.87		
	6–10 days	232	3.15	0.91		
	11–15 days	87	3.13	0.94		
	16 or more days	129	2.74	0.85		
	Total	751	3.13	0.90		
MBI-ES emotional exhaustion subscale score	Zero sick days	54	30.59	12.61	8.140	<0.001
	1–5 days	255	32.29	10.52		
	6–10 days	235	34.55	10.19		
	11–15 days	89	37.14	10.58		
	16 or more days	130	37.32	10.85		
	Total	763	34.29	10.84		
MBI-ES depersonalization subscale score	Zero sick days	54	6.96	5.71	2.542	0.039
	1–5 days	255	7.62	6.167		
	6–10 days	235	7.80	5.68		
	11–15 days	89	9.38	6.57		
	16 or more days	130	8.92	6.61		
	Total	763	8.06	6.14		
MBI-ES personal accomplishment subscale score	Zero sick days	54	35.41	8.62	2.569	0.037
	1–5 days	255	35.52	6.94		
	6–10 days	235	34.00	7.52		
	11–15 days	89	33.55	7.08		
	16 or more days	130	33.61	7.36		
	Total	763	34.49	7.37		
PSS-10	Zero sick days	54	21.04	5.90	6.66	<0.001
	1–5 days	255	21.24	6.09		
	6–10 days	235	22.50	6.055		
	11–15 days	89	24.11	6.11		
	16 or more days	130	23.78	5.55		
	Total	763	22.38	6.07		

MBI-ES, Maslach Burnout Inventory for Educators; BRS, Brief Resilience Scale; Std, Standard Deviation; *Significance at *p* ≤ 0.05. PSS-10, Perceived Stress Scale; GAD–7, Generalized Anxiety Disorder 7 Scale; PHQ-9, Patient Health Questionnaire 9 scale.

**Table 5:** LSD *post hoc* comparison of the mean difference in scores of various scales for educators who had zero sick days and those who had various sick days.

Dependent variable	(I) Sick days cat	(J) Sick days cat	Mean difference (I-J)	Sig.	95% CI	
					Lower bound	Upper bound
GAD-7 scale scores	Zero sick days	1–5 days	0.23	0.78	−1.39	1.86
		6–10 days	−0.61	0.47	−2.24	1.03
		11–15 days	−2.40^*^	0.01	−4.28	−0.52
		16 or more days	−2.32^*^	0.01	−4.08	−0.56
PHQ-9 scale scores	Zero sick days	1–5 days	0.56	0.52	−1.15	2.28
		6–10 days	−0.93	0.29	−2.66	0.79
		11–15 days	−2.24^*^	0.03	−4.21	−0.27
		16 or more days	−2.52^*^	0.008	−4.37	−0.67
BRS scores	Zero sick days	1–5 days	−0.155	0.25	−0.42	0.11
		6–10 days	0.02	0.89	−0.25	0.28
		11–15 days	0.04	0.81	−0.27	0.34
		16 or more days	0.43^*^	0.003	0.14	0.71
MBI-ES emotional exhaustion subscale scores	Zero sick days	1–5 days	−1.69	0.29	−4.82	1.44
		6–10 days	−3.96^*^	0.01	−7.11	−0.80
		11–15 days	−6.54^*^	<0.001	−10.15	−2.94
		16 or more days	−6.72^*^	<0.001	−10.11	−3.34
MBI-ES depersonalization subscale scores	Zero sick days	1–5 days	−0.66	0.47	−2.46	1.14
		6–10 days	−0.84	0.36	−2.65	0.97
		11–15 days	−2.42^*^	0.02	−4.49	−0.35
		16 or more days	−1.95^*^	0.05	−3.90	−0.01
MBI-ES personal accomplishment subscale scores	Zero sick days	1–5 days	−0.11	0.92	−2.27	2.04
		6–10 days	1.41	0.20	−0.77	3.58
		11–15 days	1.86	0.14	−0.63	4.34
		16 or more days	1.80	0.13	−0.5316	4.1310
PSS-10 scores	Zero sick days	1–5 days	−0.20	0.82	−1.9613	1.5570
		6–10 days	−1.46	0.11	−3.2330	0.3113
		11–15 days	−3.08^*^	0.003	−5.1010	−1.0497
		16 or more days	−2.74^*^	0.005	−4.6411	−0.8387

*Statistically significant mean difference.

Table 4 indicates statistically significant differences in the mean scores across all scales among respondents based on the number of sick days taken in the preceding academic year. Table 5 summarizes the *post hoc* test outcomes, which compare mean scores on the various scales between educators who had no sick days in the previous academic year and those who had sick days.

Table 4 suggests statistically significant differences in the mean scores for all the scales for respondents based on the number of sick days they had taken in the preceding academic year. Table 5 summarizes the *post hoc* test results that compare the mean scores on various scales for educators who had zero sick days in the preceding academic year and those who had various sick days.

Table 5 indicates that educators who took 11 or more sick days had significantly higher mean scores on the GAD-7 scale, the PHQ-9 scale, the PSS-10, and the MBI emotional exhaustion and depersonalization subscales than those with zero sick days. In addition, those who had 6–10 sick days showed higher mean scores on the MBI emotional exhaustion subscale than those with zero sick days. Conversely, educators who took 16 or more sick days in the preceding academic year had lower mean resilience scores, as measured by the BRS, than those with zero sick days.

## 4 Discussion

The current study assessed the number of sick days teachers took and their association with psychological issues. Our results suggest that participants who took 11 or more sick days in the preceding academic year were at least three times more likely to report high stress than those who took no sick days. The teaching profession has been associated with a higher risk of workplace stress, which can lead to burnout, anxiety, and depression (Agyapong et al., 2022). Thus, this study demonstrates that teachers who took 11 or more sick days are more likely to present with high stress in the following school year. These findings complement existing research, which suggests that stress is a major factor associated with teachers taking a leave of absence from work (Ferguson et al., 2022). A comparable study also reported a higher odds ratio for sickness absence associated with stress-related disorders (Ervasti et al., 2012).

Similarly, educators who had 11 or more sick days in the preceding academic year were at least three and a half times more likely to present with emotional exhaustion than educators with no sick days. This finding is corroborated by a study among Spanish teachers, which identified burnout (emotional exhaustion) as a significant factor associated with psychiatric sick leave among teachers (Antonio Moriana and Herruzo, 2006). Further, a recent study among educators in Canada reported a relatively higher prevalence of emotional exhaustion (Agyapong et al., 2024). Our study found no significant differences in the sense of personal accomplishment between educators with no sick days and those with sick days in the preceding year. A possible explanation is that teachers who persistently feel inadequate are more likely to leave the profession rather than return after a sickness absence. Lack of accomplishment is characterized by reduced productivity or capability, low morale, and an inability to cope (Maslach and Leiter, 2016). Parallel findings were found among Brazilian teachers, where high levels of emotional exhaustion were linked to long-term sickness absence, but low professional efficacy levels were not (Salvagioni et al., 2022). Additionally, research among Danish human service workers found that long-term sickness absence was predicted by burnout and psychosocial factors at work (Borritz et al., 2010).

The current study also found that educators with six or more sick days in the preceding academic year were at least twice as likely to present with depersonalization compared to those without sick days. Teaching, as a human service occupation, relies heavily on interpersonal interactions. Hence, feelings of cynicism and detachment from the job can severely hinder teachers’ ability to perform their duties, leading to more frequent sick absences (Maslach and Leiter, 2016). Given the high tendency of burnout (depersonalization) rate among teachers who had six or more sick days in the previous school year, this study indicates that this cohort of teachers is at an increased risk of taking additional sick days in the current school year. Furthermore, teaching entails specific skills and training, making it challenging to replace experienced educators (Cieslinski and Szum, 2014). Therefore, it is vital for these teachers to be supported to reduce the tendency for increased sick leaves.

The current study found that teachers who had 11–15 sick days in the preceding academic year were three times more likely to present with likely GAD compared to those who had no sick days. A report by general practitioners on cases of work-related ill-health found that 81% of psychological issues resulted in sickness absence (Hussey et al., 2012). Sick days may also serve as a means of escaping work-related anxiety for workers who struggle with participation in work, resulting in likely long-term sick leave (Muschalla, 2016). A regression analysis in another study identified anxiety as the sole predictor for the length of sickness absence (Schneider et al., 2017). Additionally, research has shown that job-related anxiety significantly correlates with the duration of sick leave (Muschalla et al., 2010). Thus, teachers who took 11–15 sick days in the preceding school year may be at an increased risk of taking additional sick days in the current school year due to the higher prevalence of anxiety in this group.

Similarly, teachers with 11 or more sick days in the preceding academic year were about three times more likely to present with likely MDD than those without sick days. Consistent with this finding, a study reported moderate evidence linking more severe depressive disorders with a history of previous sick leave (Lagerveld et al., 2010). A systematic review and meta-analysis also found that depressive symptoms were associated with sick leave (Amiri and Behnezhad, 2021). Additionally, a Dutch database showed that burnout, anxiety, and depression disorders are associated with longer sick leave durations, particularly among educators (Flach et al., 2012). Similar findings were reported by a U.K. study, which showed an association between sickness absence and depressive symptoms (Kidger et al., 2016). Given the high prevalence of likely MDD among teachers with 11 or more sick days in the previous school year, this study indicates that these teachers may be at an increased risk of taking additional sick days in the current school year.

With respect to resilience, this study found that teachers who had 16 or more sick days in the preceding academic year were about three times more likely to present with low resilience compared to those with no sick days. Teachers with low resilience are more susceptible to other psychological problems, such as stress and burnout, which can lead to breakdowns and increased sick leave. Research has shown that a growing number of teachers are experiencing higher stress and workload, negatively impacting their physical and mental well-being (British and Columbia’s Teachers' Federation, 2023). Further, a recent study in Canada found that educators with low resilience were 3.26 times more likely to experience symptoms of emotional exhaustion (Agyapong et al., 2024), which has also been implicated in mental health-related sickness absence among Spanish school teachers (Antonio Moriana and Herruzo, 2006). Thus, it can be inferred that teachers who had 16 or more sick days in the preceding school year may be at an increased risk of taking additional sick days in the current school year due to their low resilience levels.

The results of this study suggest that educators who had 11 or more sick days had significantly higher mean scores on the PSS-10, the MBI Emotional Exhaustion subscale, and the MBI Depersonalization subscale, the GAD-7 scale, and the PHQ-9 scale compared with those who had no sick days. In addition, those who took 6–10 sick days had a higher mean score on the MBI Emotional Exhaustion subscale than those with no sick days. Emotional exhaustion is believed to develop first in response to high job demands and is predictive of stress-related health outcomes such as weariness, loss of energy, depletion, debilitation, and fatigue (Maslach and Leiter, 2016). These results are expected, as teachers who take more sick days are likely to have increased vulnerability to developing or already having established psychological issues. Existing literature also indicates that anxiety, depression, emotional exhaustion, and depersonalization are risk factors for increased sick leave among educators (Ferguson et al., 2022; Toppinen-Tanner et al., 2005). Thus, the findings from this study support the proposition that there is an increased likelihood that educators who took six or more sick days in the previous school year are more likely to take additional sick days in the current school year due to ongoing mental health problems.

Resilience emerges from a dynamic interplay between individual risk and protective factors, enabling teachers to persevere and thrive in the face of challenges (Beltman et al., 2011). Consistent with this, our study shows that educators with 16 or more sick days in the preceding academic year had lower mean scores on the Brief Resilience Scale (BRS) than those with zero sick days. Another study found an inverse relationship between resilience and depressive symptoms, where higher resilience correlated with lower Beck Depression Inventory scores, indicating reduced burden from depressive symptoms (Engmann, 2013). Hence, our study findings also support the proposition that there may be an increased likelihood that educators who took more sick days in the preceding school year will take additional sick days in the current school year due to low resilience and vulnerability to ongoing mental health problems.

### 4.1 Study limitations

First, although standardized, the scales used in assessing educators’ psychological issues are not meant to be diagnostic. Thus, the findings may not accurately reflect the prevalence of diagnosed psychological disorders among educators but provide an indication of psychological distress among the educators. Future research may explore incorporating structured clinical assessments conducted by qualified professionals to provide a more accurate and comprehensive understanding of the mental health challenges among educators and their association with sick days. Second, the study was undertaken in three provinces of Canada, and therefore, the findings may not be generalizable to all educators in Canada. The socio-economic contexts can vary significantly across provinces, and these differences could influence the psychological well-being of educators. Future studies should also aim to conduct similar research in other provinces of Canada, aside from the provinces’ understudy, to determine if similar outcomes would be established. A significant proportion (over 80%) of the study participants were female. Although there is generally gender imbalance in the teaching profession, with females disproportionately represented, this percentage may not be reflective across the three provinces. Thus, the demographic variables may not reflect the demographics of teachers in the three provinces under study, and therefore, the findings may not be generalizable. Third, the survey questions about educators’ sick days were not validated and relied on self-reported data, which may not accurately capture the actual sick days taken during the academic year. Notwithstanding these limitations, this study provides valuable insights into the relationship between sick days and psychological issues among educators, which would be of interest to policymakers and could inform policies in the field of education. This study also adds to the limited Canadian literature that explores the relationship between teacher sick days and mental health.

## 5 Conclusion, implications for policy, and future research

This study has demonstrated a clear link between the number of sick days taken by teachers and the prevalence and severity of stress, certain aspects of burnout, low resilience, anxiety, and depression. Existing research also indicates that these psychological issues are associated with increased sick days. Therefore, it is reasonable to suggest that teachers who reported sick days in the preceding school year are at an increased risk of taking additional sick days in the current school year due to the increased psychological burden among these educators. In order to enhance the reliability of self-reported data, future Canada wide studies should use mixed qualitative and quantitative methods, including structured clinical interviews to aid diagnostic accuracy and focus group discussions which could provide better insights into the association between psychological problems and sick days in educators.

Clearly, this inference needs to be validated through further research. Despite the need for additional studies, this research suggests that teachers who take sick days, regardless of the reason, should be offered interventions addressing psychological issues as a preventative measure to reduce the likelihood of further sick leave. Preserving teachers’ psychological well-being is crucial for their effective performance and for supporting the overall educational experience of students, including their academic success and personal and social well-being. Short-term sickness absences can escalate to long-term absences if teachers are not adequately supported. Implementing workplace health promotion initiatives can also play a significant role in addressing mental health challenges among educators and enhancing their overall psychological wellness, which would, in turn, help to ensure that students attain their full potential. Additionally, interventions that focus on promoting self-compassion, with the possibility of increasing positive affect and reducing negative affect, could have a positive impact on both current and future resilience (Eryılmaz et al., 2024).

Consequently, building on these findings, future research can help develop more effective strategies for supporting educators’ well-being, ultimately improving outcomes for teachers and students. Governments and policymakers in the education sector should endeavor to create a supportive environment that enables teachers to thrive in their professional roles without resorting to increased sick leave, which can jeopardize student learning and achievement.

## Data Availability

The raw data supporting the conclusions of this article will be made available by the authors, without undue reservation.
